# Combining diffusion and transformer models for enhanced promoter synthesis and strength prediction in deep learning

**DOI:** 10.1128/msystems.00183-25

**Published:** 2025-03-19

**Authors:** Xin Lei, Xing Wang, Guanlin Chen, Ce Liang, Quhuan Li, Huaiguang Jiang, Wei Xiong

**Affiliations:** 1 School of Future Technology, South China University of Technology 26467 , Guangzhou, Guangdong, China; 2 School of Biology and Biological Engineering, South China University of Technology 199191 , Guangzhou, Guangdong, China; 3 Research Projects Department, Guangdong Artificial Intelligence and Digital Economy Laboratory (Guangzhou) 651386 , Guangzhou, Guangdong, China; Florida Atlantic University, Boca Raton, Florida, USA

**Keywords:** deep learning, diffusion model, synthetic promoter, transformer

## Abstract

In the field of synthetic biology, the engineering of synthetic promoters that outperform their natural counterparts is of paramount importance, which can optimize the expression of exogenous genes, enhance the efficiency of metabolic pathways, and possess substantial commercial value. Research indicates that some synthetic promoters have higher transcriptional activity compared to strong natural promoters. However, with the exponential increase in complexity due to the 4^*n*^ potential combinations in a promoter sequence of length *n*, identifying effective synthetic promoters remains a formidable challenge. Deep learning models, by adaptively learning from extensive data sets, have become instrumental in analyzing biological data. This study introduces a diffusion model-based approach for designing promoters viable in model bacteria such as *Escherichia coli* and cyanobacteria. This model proficiently assimilates and utilizes inherent biological features from natural promoter sequences to engineer synthetic variants. Additionally, we employed a transformer model to evaluate the efficacy of these synthetic promoters, aiming at screening those with high performance. The experimental findings suggest that the synthetic promoters by the diffusion model not only share key biological features with their natural counterparts but also demonstrate greater similarity to natural promoters than those generated by a variational autoencoder. In predicting promoter strength, the transformer model demonstrated improved performance over the convolutional neural network. Finally, we developed an integrated platform for generating promoters and predicting their strength.

**IMPORTANCE:**

We demonstrated that diffusion models are superior in accomplishing the promoter synthesis task compared to other state-of-the-art deep learning models. The effectiveness of our method was validated using data sets of *Escherichia coli* and cyanobacteria promoters, showing more stable and prompt convergence and more natural-like promoters than the variational autoencoder model. We extracted sequence information, dimer information, and position information from promoters and combined them with a transformer model to predict promoter strength. Our prediction results were more accurate than those obtained with a convolutional neural network model. Our *in silico* experiments systematically introduced mutations in promoter sequences and explored their contribution to promoter strength, highlighting the depth of learning in our model.

## INTRODUCTION

Promoters are essential regulatory elements that modulate the transcriptional activity of specific genes (1, 2). Utilizing high-performance promoters offers several advantages: enhancing gene synthesis efficiency (3, 4), shorter durations of biological processes (5), and lower biomanufacturing costs (6). Significantly, a library of high-performance synthetic promoters holds substantial commercial potential due to its intellectual property advantages over its natural counterparts. Thus, the discovery of high-performance promoters is critical for advancements in synthetic biology (7). Nevertheless, the efficacy of promoters is typically constrained, and their screening and optimization demand considerable time and resources, which poses significant challenges to the field of synthetic biology (8). Consequently, the quest for high-performance synthetic promoter sequences has become a focal point in the era of synthetic biology (9). Modifying and optimizing natural promoters are common strategies for developing high-performance synthetic promoters (10, 11). For instance, to significantly boost the efficiency of extracting plant essential oils, Park et al. (12) generated a variety of synthetic promoters by randomly mutating natural ones and identified high-performing ones using fluorescence-activated cell sorting. Similarly, Han et al. (13) identified key regulatory regions within promoter sequences and conducted two rounds of mutagenesis in these areas to generate new promoters. By measuring green fluorescent protein fluorescence intensity and mRNA expression levels through RT-qPCR, the researchers accurately assessed the transcriptional strength of mutant promoters and successfully synthesized high-strength promoters with triple the activity of natural ones. Despite the satisfactory performance and simplicity of these methods, they have exhibited some limitations. Specifically, mutating only parts of natural promoters restricts the exploration of the exponentially growing promoter sequence space, thereby hindering the discovery of high-performance synthetic promoters. Given a promoter length of *n*, the search space encompasses 4^*n*^ combinations. It is a formidable challenge for researchers to isolate high-performance synthetic promoters in an expansive search.

In recent years, deep learning methods have been extensively applied to protein structure prediction (14–16), drug development (17, 18), and medical image analysis (19). Deep generative models, such as the generative adversarial network (GAN) and variational autoencoder (VAE), have demonstrated great potential for the task of synthesizing promoters. For instance, Wang et al. (20) effectively synthesized *Escherichia coli* promoters using a GAN and confirmed their biological activity through wet experiments. Seo et al. (21) generated tens of thousands of cyanobacterial promoters using a VAE and successfully validated their biological activity, indicating that VAE is capable of capturing key features of cyanobacterial promoters. These studies have highlighted the significant potential of deep learning in designing high-performance synthetic promoters and have indicated the effectiveness through statistical analyses and biological experiments. However, notable gaps still exist. Specifically, the complexity of current deep learning approaches (e.g., GAN) makes the training task a grand challenge. In addition, comprehensive insights into how deep generative models process biological features remain limited. The absence of detailed explanations obscures the underlying mechanisms these models employ in synthesizing promoters. A thorough understanding of how deep generative models integrate biological features is crucial for further improving the design efficiency and effectiveness of synthetic promoters.

Diffusion models, leveraging diffusion theory and the Markovian property, have become widely used in text and image generation, attributed to their stable training processes and rapid convergence speeds (22–24). These models operate in two distinct stages: noise addition and noise removal. In the noise addition stage, known noise is incrementally introduced to alter the original data distribution. Subsequently, during the denoising stage, the model progressively predicts and removes the noise, reconstructing the original data. Through this process, diffusion models can analyze the latent feature distribution within the data set, accurately predict noise, and generate new samples that reflect the data set’s feature distribution through denoising. Researchers have pioneered diffusion models in the fields of chromosome regulation and gene expression (25). A diffusion model was employed to design context-specific DNA regulatory sequences (26), demonstrating significant potential for novel therapeutic applications that demand precise gene expression regulation. Moreover, diffusion models have been employed to predict the colony growth trend (27) and generate medical images (28). These studies demonstrate that diffusion models possess substantial potential for handling biological data and offer innovative tools and methodologies for the field of synthetic biology.

The transformer model (29–31), initially proposed by Google researchers in 2017, has been extensively applied across various domains, such as data forecasting (32), image recognition (33), and text generation (34, 35). The transformer model, consisting of multiple encoders and decoders, utilizes a self-attention mechanism to capture sequence features effectively. Researchers have utilized the transformer model for classifying promoters in *E. coli* (36). Given its proficiency in handling long sequences, the model has also been used to identify enhancer-promoter interactions and predict target genes for non-coding mutations (37). Furthermore, the transformer model plays a significant role in predicting transcription start sites (38) and gene expression levels (39). These studies inspired us to explore the vast potential of this model in the scenario of predicting synthetic promoter strength.

This article presents a promoter design strategy using a diffusion model and screens high-performance promoters through a transformer-based prediction model. First, a diffusion model is trained using promoter data sets to extract the biological features of natural promoters and generate numerous synthetic promoters. Next, a transformer-based promoter strength prediction model is developed to identify high-performance synthetic promoters. Compared to VAE, the promoters created by diffusion models exhibit superior performance in metrics, such as the position distribution of 6-mer frequencies, sequence logos, and k-mer frequency correlations. Furthermore, the training process is found to be stable, as evidenced by the consistent convergence of the loss function and the absence of large fluctuations in model performance during training. A detailed demonstration of the biological feature learning process in deep generative models is provided. Compared to the convolutional neural network (CNN), the transformer-based model extracts multifaceted features related to promoter strength, including sequence and dinucleotide information, resulting in enhanced prediction performance.

## MATERIALS AND METHODS

### Promoter data sets

This study utilized a data set of *E. coli* K12 MG1655 natural promoters containing 11,884 samples, with each sample comprising a 50-nt promoter sequence and its corresponding strength (20). Most promoters in this data set consist of sequences extending 50 bp upstream from the transcription start site, with promoter strength determined via dRNA-seq, as documented by Thomason et al. (40). Additionally, to extend the validation of the diffusion model to other prokaryotic systems, a separate promoter data set from *Synechocystis* sp. PCC6803 was also utilized (21). The natural promoter sequences of cyanobacteria were obtained by extracting the 100 bp region upstream of the open reading frame. The strength of these natural promoters was similarly determined by expression levels measured via dRNA-seq. In the promoter strength prediction task, the encoded promoter sequence serves as the model’s input feature, and promoter strength measured via dRNA-seq was used as the output label. The transformer-based prediction model is trained using supervised learning. Conversely, the promoter generation task is conducted as an unsupervised process, where only promoter sequence information is utilized by the diffusion model. Details regarding two natural promoter data sets are available in Files S1 and S2 , respectively.

### Data processing

**Fig 1:**
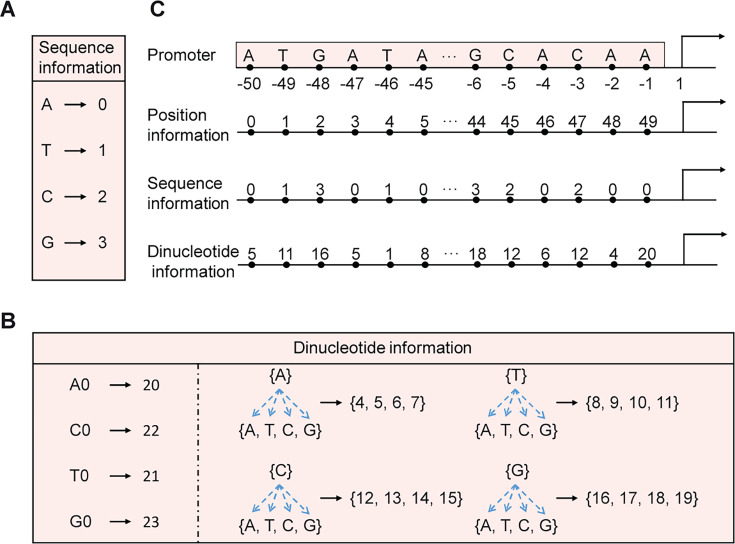
Encoding methods for input features of the prediction task. (**A**) The encoding method for sequence information. The nucleotides A, T, C, and G are represented by the integers 0–3, respectively. (**B**) The encoding method for dinucleotide information. Combinations of group A, T, C, and G with group A, T, C, and G are represented by the integers 4–19, respectively. To ensure the dinucleotide information, sequence information, and positional information have the same dimensions, zero padding is applied at the end of the promoter sequence, resulting in the dinucleotide combinations A0, T0, C0, and G0. These combinations correspond to the integers 20–23. (**C**) An example of this encoding method.

Diverse encoding methods were utilized to process promoter sequences, tailored to the specific requirements of each task. One-hot encoding was applied in the promoter design task. To predict promoter strength, sequence, dinucleotide, and positional information were encoded to enrich the input of the predictive model. These encoding techniques are depicted in Fig. 1. Such varied encoding strategies ensure ample data support for different applications, thereby improving the performance of both generative and predictive models.

In promoter strength prediction, normalization of strength values is critical. Promoter strength data typically exhibits a skewed distribution, with most values concentrated in the low-strength range and a few in the high-strength range. Direct normalization of skewed data can compress the majority of values into a narrow range while retaining large magnitudes for outliers. This uneven distribution can reduce the model’s learning efficiency and prediction accuracy, as the model may overemphasize extreme values and overlook regular data points. Applying a logarithmic transformation compresses the data’s dynamic range, facilitating subsequent analysis. Therefore, in this study, we first apply a logarithmic transformation to the raw data, followed by normalization.


(1) $$X_{\text{log}}=\log_{10}(X).$$

*X*_log_ represents the log-transformed promoter strength data with base 10, and *X* represents the original promoter strength data.

(2) $$X_{\text{norm}}=\frac{X_{\text{log}}-\min(X_{\text{log}})}{\max(X_{\text{log}})-\min(X_{\text{log}})}.$$

*X*_norm_ represents the nomalized promoter strength data, min(*X*_log_) is the minimum value of the log-transformed data, and max(*X*_log_) is the maximum value of the log-transformed data.

### Diffusion model

**Fig 2:**
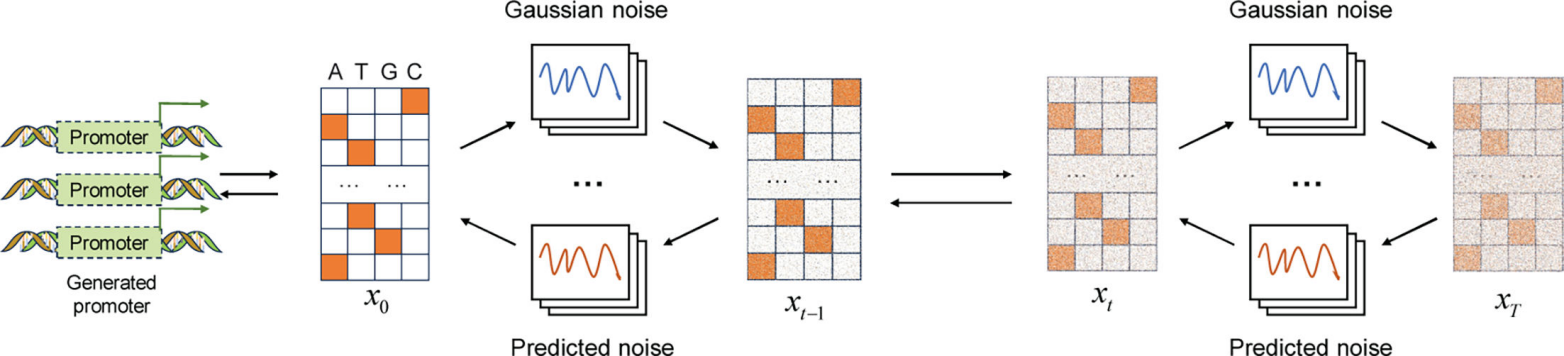
The framework of the diffusion model for promoter generation.

The denoising diffusion probability model (DDPM) comprises two primary components: noise addition and denoising (25, 41). Both components adhere to the Markovian property, which implies that the current state in both the noise addition and denoising processes only depends on the previous state, not on any earlier states. As illustrated in Fig. 2, during the noise addition process, DDPM utilizes a conditional distribution to incrementally introduce specific Gaussian noise into the original data *x*_0_, which represents one-hot encoded promoter sequences. *T* signifies the total number of noise addition steps. A larger *t* corresponds to more noise being added, where *t* ∈ {1,2,...,*T*} . Consequently, after *T* steps, the original data *x*_0_ are converted into Gaussian noise data *x*_*T*_. Owing to the Markovian property of the noise addition process, *x_t_* depends only on its immediate predecessor *x*_*t-1*_ and the corresponding noise introduced at that step. In the denoising phase, the model predicts and eliminates noise from the data based on a learned conditional distribution, thereby reconstructing the promoter data from the Gaussian noise. This study involves training DDPM with two one-hot encoded promoter data sets and subsequently synthesizing promoter sequences through its denoising mechanism.

### Transformer

**Fig 3:**
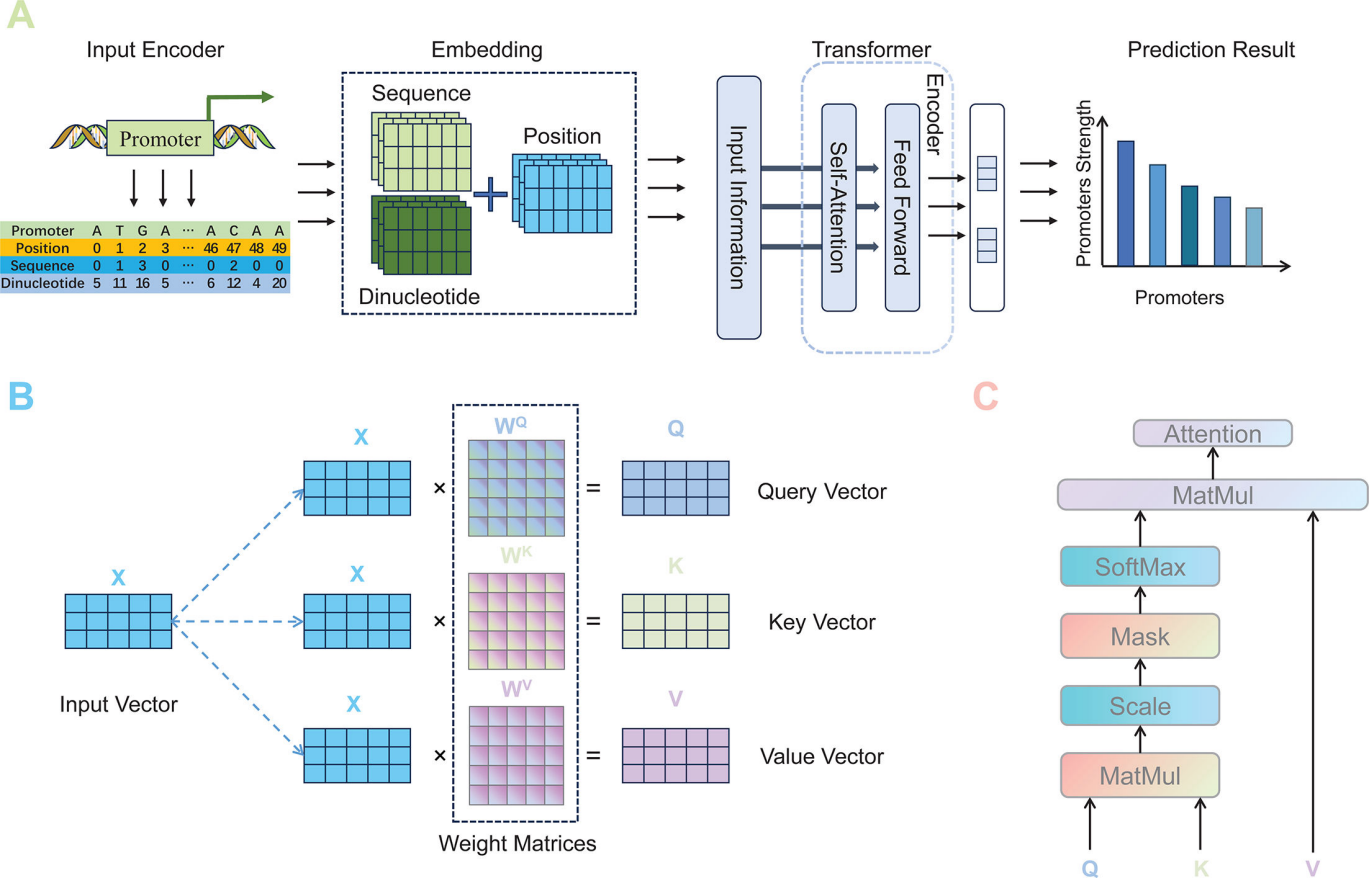
Transformer-based prediction method of the promoter strength. (**A**) Input encoding method and model structure. The input encoder converts nucleotide sequences and positional information into numerical embeddings, which capture the structural characteristics of the sequence. These embeddings are processed by the transformer encoder through self-attention and feed-forward layers to generate a sequence representation. Finally, the model outputs the predicted strength of each promoter. (**B**) Generation of query, key, and value vectors. The input vector *X* is projected into three distinct weight matrices *W*^*Q*^, *W*^*K*^, and *W*^*V*^ simultaneously to generate the query, key, and value vectors. They capture similarity and relationships between different positions within the sequence. (**C**) Computation flow of self-attention coefficient. First, the dot product of the query and key vectors is scaled to stabilize the gradients. An optional mask is then applied to control which positions participate in the computation. Next, a Softmax operation is applied to the similarity scores to produce weights. These weights are subsequently multiplied by the value vector to generate the attention output representation.

As depicted in Fig. 3, this study utilizes a transformer-based method to predict promoter strength. At the heart of the prediction task lies the transformer architecture, characterized by an encoder framework: this component consists of multiple layers, each featuring self-attention mechanisms and feed-forward neural networks. The self-attention mechanisms enable the model to assess the significance of various positions within the promoter sequence, thereby enhancing the dynamic comprehension of the sequence’s contextual relationships. Employing this transformer-based methodology capitalizes on the intricate patterns in genetic data, significantly enhancing the precision and effectiveness of promoter strength predictions over traditional methods like CNN.

### Pearson correlation coefficient

The Pearson correlation coefficient (PCC) is a statistical metric that quantifies the strength and direction of the linear relationship between two variables *X* and *Y*. The formula for its calculation is as follows:


(3) $$PCC(X,Y)=\frac{\sum_{i=1}^{n}(X_{i}-\bar{X})(Y_{i}-\bar{Y})}{\sqrt{\sum_{i=1}^{n}(X_{i}-\bar{X}{)}^{2}\sum_{i=1}^{n}(Y_{i}-\bar{Y}{)}^{2}}},$$

where *X_i_* and *Y_i_* are the *i*th values in the data sets *X* and *Y*, respectively; $\bar{X}$ and $\bar{Y}$ are the means of the data sets *X* and *Y*; and *n* is the total number of data points.

The PCC ranges from −1 to 1, where values near 1 indicate a strong positive correlation, and values near −1 indicate a strong negative correlation. In deep learning, PCC serves not only as a metric for evaluating model performance but also as a loss function for optimization, particularly in tasks where a strong linear correlation between model outputs and true labels is required. In this study, we adopt PCC as a metric to assess the performance of both generative and predictive models. Furthermore, for the predictive task, we combine PCC with mean squared error to form the loss function that guides model training.

## RESULTS

### The computational framework for promoter generation and strength prediction

**Fig 4:**
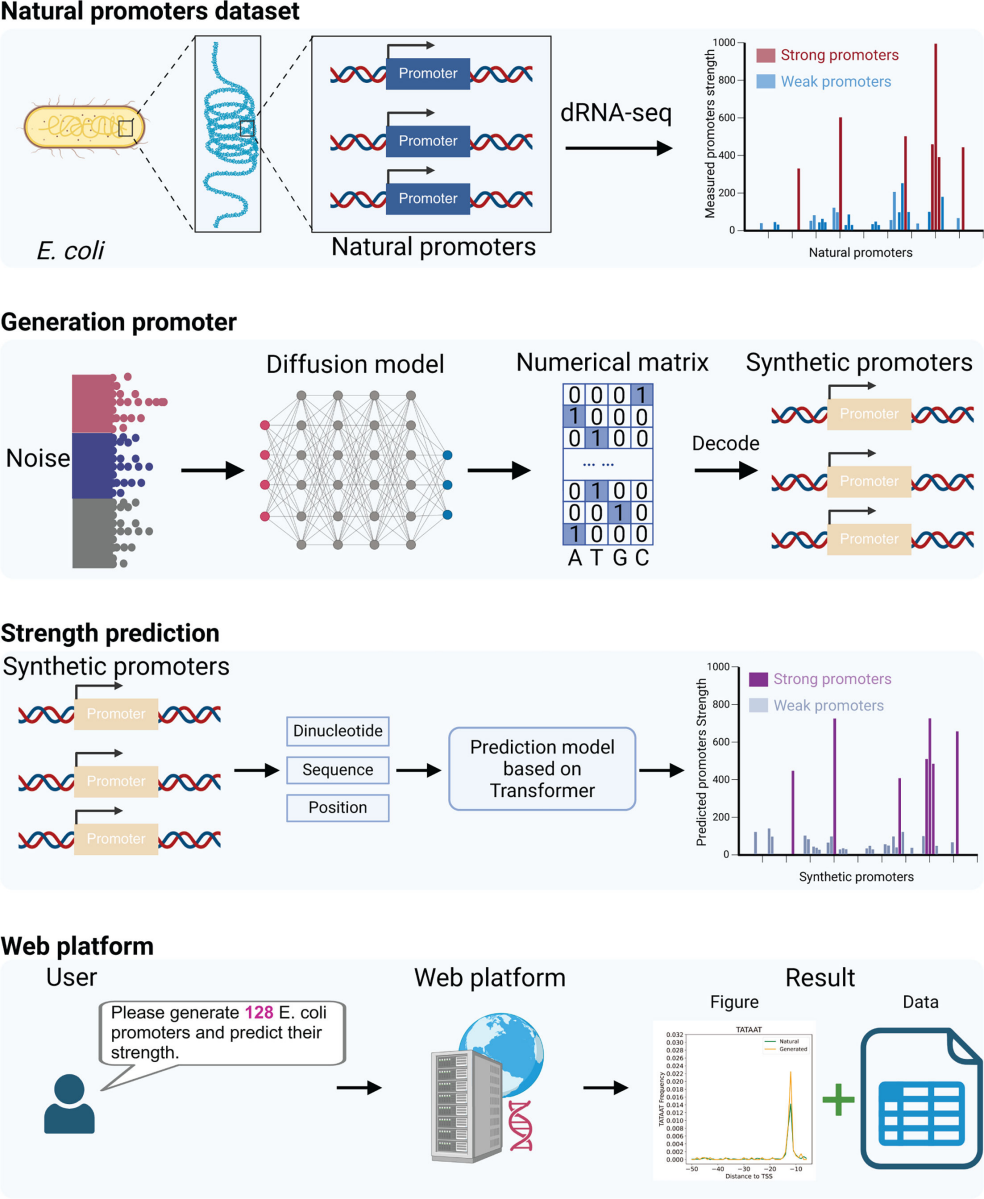
Schematic diagram of the experimental framework. In the first step, this study compiles a natural promoter data set comprising promoters and their strengths measured by dRNA-seq reported in previous research. Then, the diffusion model is trained to generate a large number of synthetic promoters. Next, a transformer-based predictive model is trained to screen for strong synthetic promoters. Finally, we have integrated the trained prediction and generation models into a web platform to facilitate usage by researchers without a background in deep learning.

Figure 4 provides an overview of the technical workflow, which primarily comprises three phases: data set, promoter generation, and promoter strength prediction. In the first step, we obtained a data set of natural promoters from previous studies. This data set contains two key types of information: natural promoter sequences and promoter strength measured by dRNA-seq, which were used to train the subsequent generation and prediction models. As the next move, we trained a diffusion model for generating promoters. Using the natural promoter data set, we trained the diffusion model in an unsupervised manner. The promoter sequences were one-hot encoded to convert them into a numerical format compatible with the diffusion model. After training, the diffusion model could generate numerical matrices with promoter characteristics from noise, which were then decoded into promoters. Following this, we used the trained transformer-based model to identify high-performance synthetic promoters. Details of the prediction model’s training are provided in Fig. 3. Given the large number of synthetic promoters, performing biological validation on all of them would be time-consuming. Therefore, the prediction model was employed to screen high-performance candidates from the generated promoters for experimental validation by biologists. The specific approach involves extracting the sequence, dinucleotide, and positional information of synthetic promoters as input features. The predictive model analyzes these features to map them to the corresponding strength information. In the end, the workflow is fully integrated into the https://bioinformatics-syn.org/ promoter design platform in this work. Users can access the platform, specify the desired number of promoters, and receive synthesized promoter sequences along with their predicted strength. Our platform visualizes the distribution characteristics of synthetic and natural promoters and allows the results to be downloaded in Excel format.

### Synthetic *E. coli* promoters based on the diffusion model

**Fig 5:**
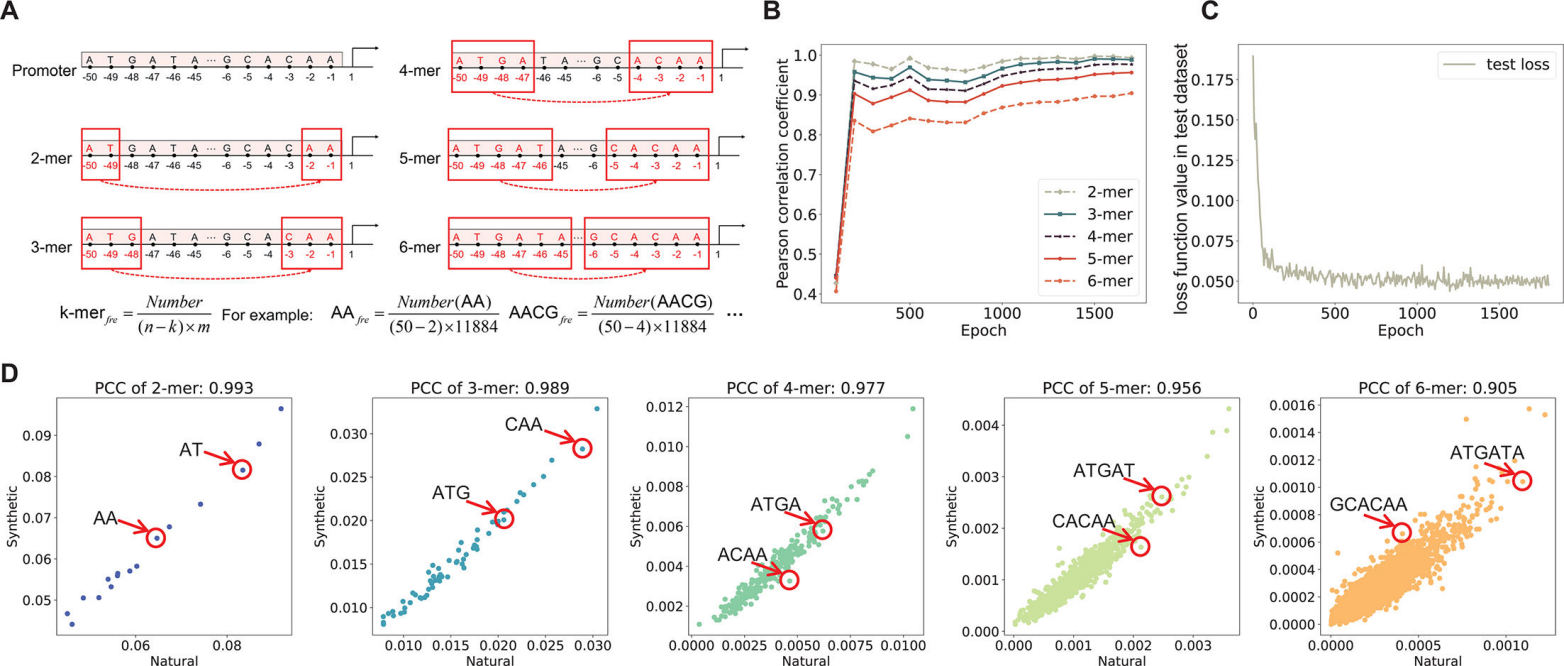
The performance of synthetic promoters. (**A**) The method for calculating k-mer frequency in promoters. For a given promoter sequence, a sliding window of length *k* is used with a step size of one nucleotide to count the occurrences of various k-mers. The k-mer frequency for the entire promoter data set is then calculated using a specific formula, where *n* is the length of the promoter sequence, *m* is the number of promoters in the data set, and Number represents the occurrences of a particular k-mer across all promoter sequences in the data set. (**B**) The trend of k-mer frequency correlation between synthetic and natural promoters during training helps us evaluate the fidelity of synthetic promoters in preserving the k-mer structure of natural promoters. (**C**) During the training of the diffusion model, changes in the loss function values on the test set were monitored. The loss function used was the absolute error (L1 loss). The loss decreases sharply during the initial epochs and then stabilizes at a low level, indicating that the model has converged without signs of overfitting. The stability and low final loss values further demonstrate the model’s robustness in generating promoters. (**D**) The scatter plot shows the k-mer (*k* = 2–6) frequency correlation (PCC) between synthetic and natural promoters. Each point represents the frequency of a specific k-mer element (e.g., “AA,” “ATG,” “ATGA,” “ATGAT,” and “ATGATA”) in natural promoters (*x*-axis) and synthetic promoters (*y*-axis).

**Fig 6:**
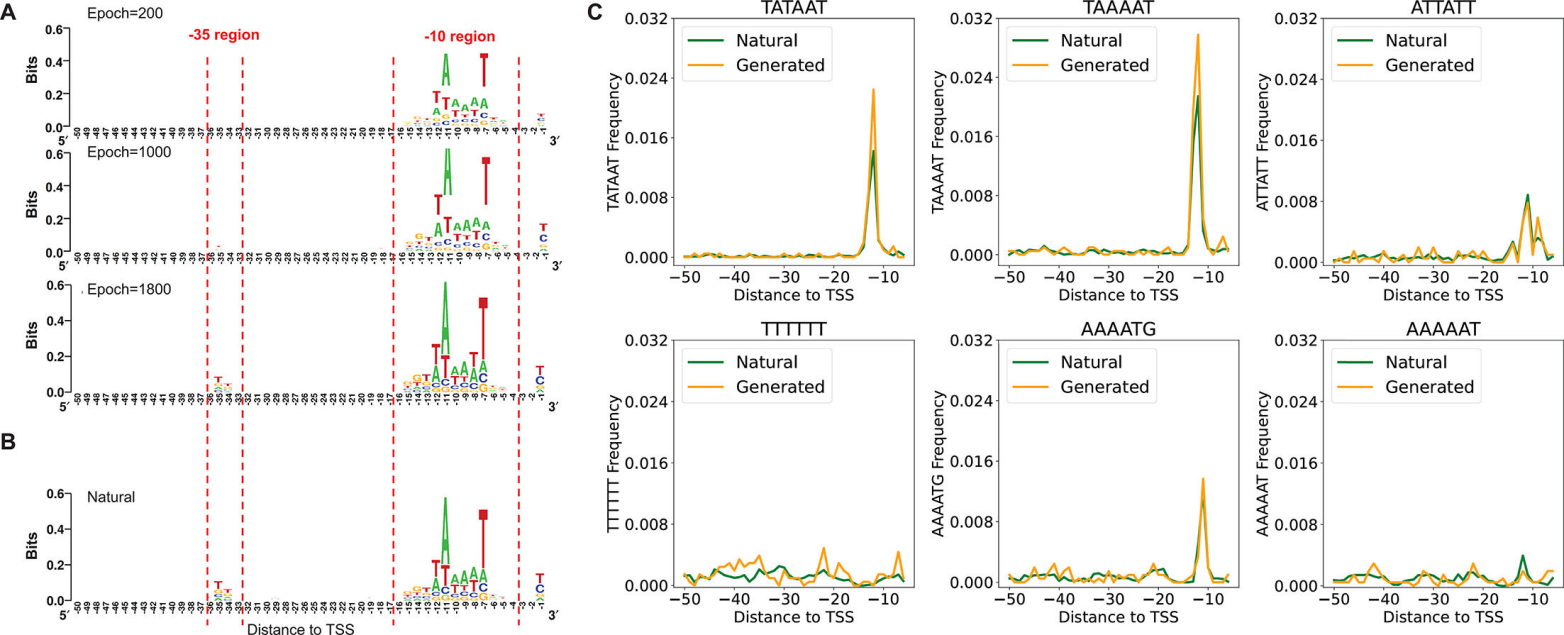
The sequence logo of synthetic and natural promoters and position distribution of 6-mer frequency. (**A**) Sequence logos of synthetic promoters at different stages of training. (**B**) Sequence logo of natural promoters. (**C**) Position distribution of the six 6-mer elements’ frequencies of synthetic and natural promoters.

First, this study recorded the biological characteristics of synthetic promoters generated by the DDPM throughout the training process, with detailed results depicted in Fig. 5. As illustrated in Fig. 5A, k-mer occurrence in promoters was calculated for quantitative evaluation of the learning process with respect to promoter synthesis. Figure 5D illustrates the correlations of k-mer frequency, where points are largely distributed around the “*y* = *x*” line, suggesting that k-mer frequencies are nearly identical between natural and synthetic promoters. Scatter plots of k-mer frequency correlations at various training stages are available in File S3. Figure 5B illustrates the PCC between k-mer frequencies of synthetic and natural promoters across different training stages. Initially, the correlation increases rapidly within the first 200 epochs and then the PCC values stabilize, reflecting a swift learning process. Although the PCCs for 2-mer to 6-mer frequencies gradually decrease, they consistently remain above 0.89. High PCC indicates that the generated promoters exhibit statistical characteristics similar to natural promoters, demonstrating the effectiveness of our diffusion model in synthesizing promoter tasks. Furthermore, we analyzed the synthetic promoters using sequence logos and position distribution of 6-mer frequency. Sequence logos were generated for both synthetic promoters (Fig. 6A) and natural promoters (Fig. 6B). Our findings indicate that the trained DDPM successfully captured the sequence motifs in the −10 and −35 regions of natural promoters. Initially, prior to reaching 1,000 epochs, the DDPM predominantly focused on the sequence motifs in the −10 region, while those in the −35 region appeared more random. However, after surpassing 1,000 epochs, the model progressively mastered the motifs in the −35 region. This trend likely stems from the clearer sequence conservation of the −10 region in natural promoters (Fig. 6B), which facilitates easier learning by the model. Regarding the position distribution of the 6-mer frequency metric, we visualized the frequencies of “TATAAT” and the five other most frequent 6-mer elements of natural promoters in Fig. 6C (20). The analysis reveals a striking similarity in the positional distribution of these six 6-mers between natural and synthetic promoters, indicating that the diffusion model successfully captures and replicates the essential sequence characteristics of natural promoters.

### Analysis of synthetic cyanobacterial promoters based on the diffusion model

**Fig 7:**
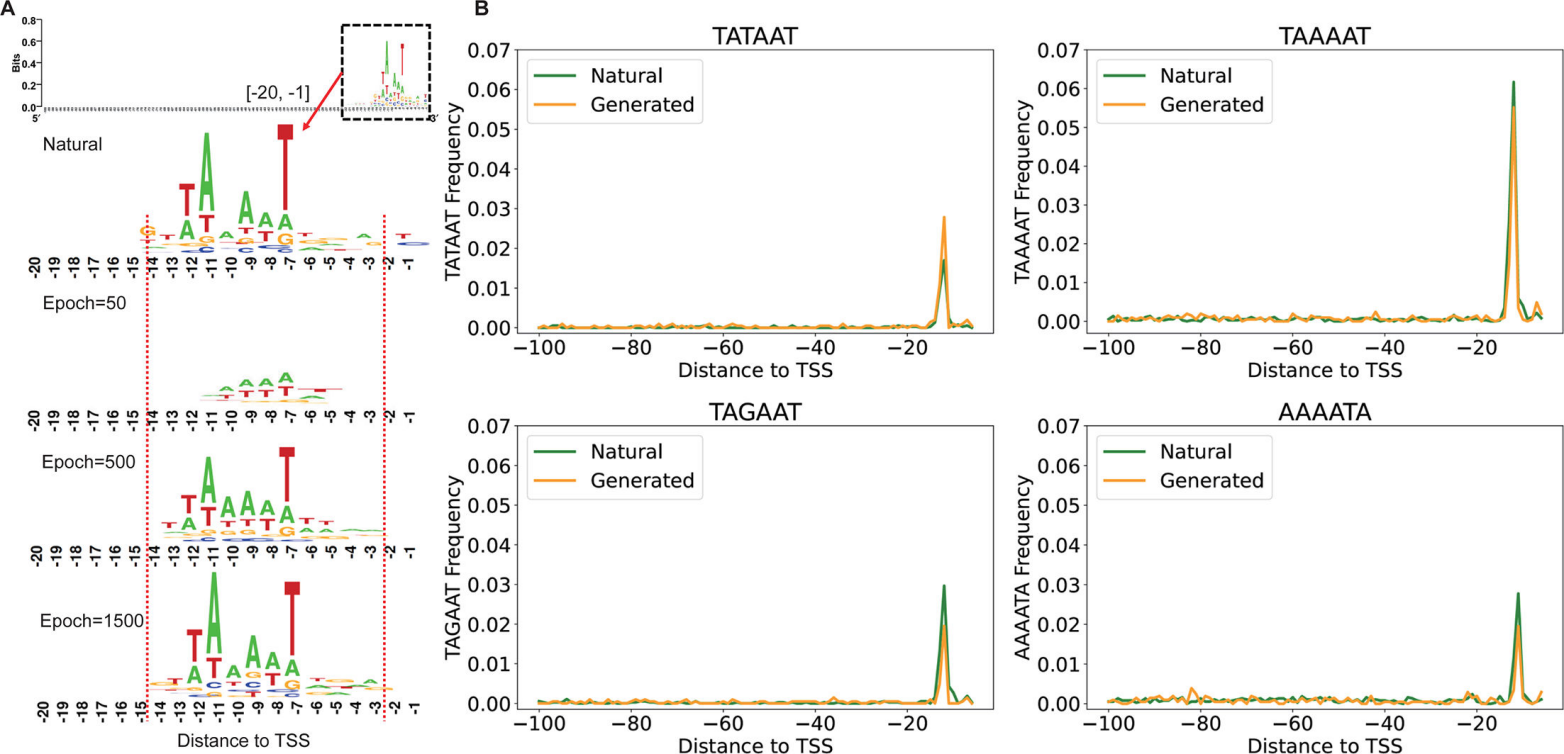
The results of the cyanobacterial promoter synthesis experiment. (**A**) Sequence logos for the [−20, −1] region of natural and synthetic promoters. (**B**) Position distribution of 6-mer frequency for natural and synthetic promoters.

To validate the robustness of the DDPM in synthesizing promoters irrespective of the training data set, we tested an additional data set from the cyanobacterium *Synechocystis* sp. PCC6803 (21), with the specific results illustrated in Fig. 7. As depicted in Fig. 7A, the similarity between the synthetic and natural promoter sequence logos increases with ongoing training. Although the model fails to capture the sequence characteristics at the −1 position. In the [−14, −2] region, synthetic and natural promoters exhibit highly similar sequence features. Moreover, the consistency of sequence conservation between synthetic and natural promoters is demonstrated (Fig. 7B). Notably, the performance of the DDPM exceeds that of the recently reported VAE model. The synthetic promoters generated by the VAE model (21) show positional discrepancies in the distribution of the “TAGAAT” motif frequency compared to natural promoters. In contrast, these discrepancies are absent in the DDPM. These observations imply that the DDPM not only adapts more effectively to two promoter data sets but also shows a possible advantage over the VAE model in capturing essential biological features of cyanobacterial promoters.

### *E. coli* promoters synthesized by the DDPM model versus VAE

**Fig 8:**
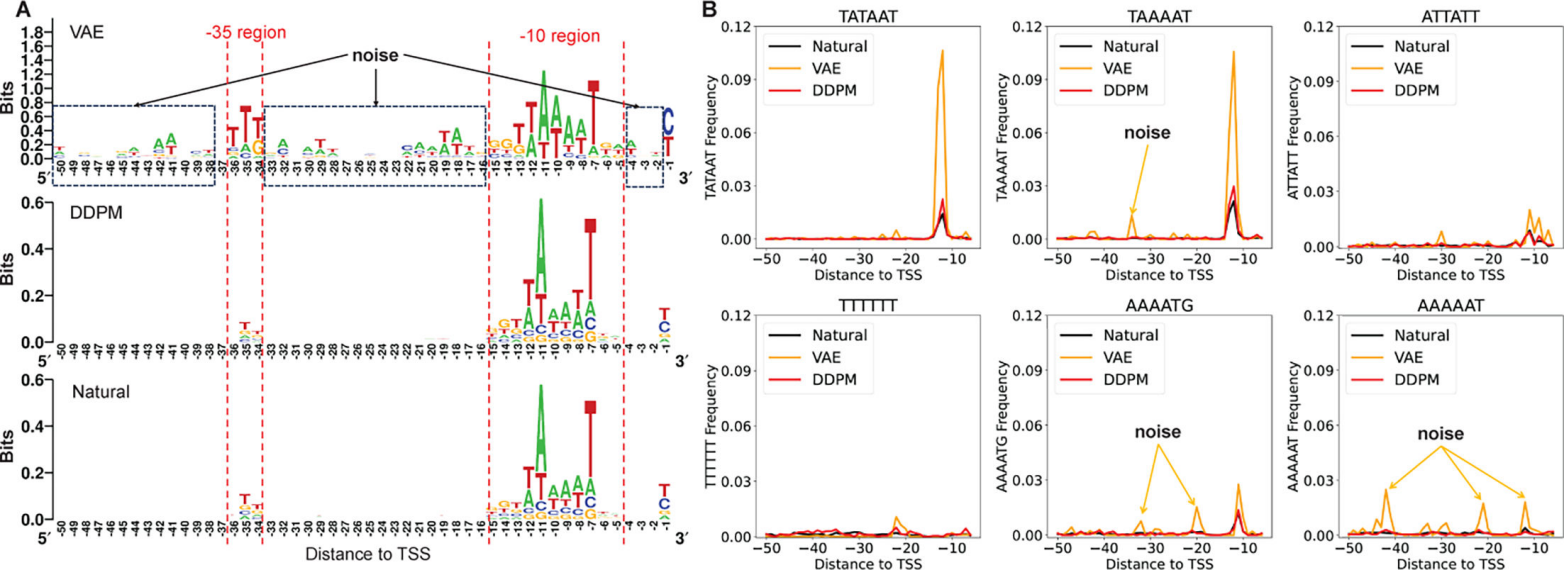
The performance of VAE and DDPM in the *E. coli* promoter synthesis task. (**A**) Sequence logos of promoters generated by DDPM, VAE, and natural promoters. (**B**) The position distribution of 6-mer frequency for natural promoters and promoters generated by DDPM and VAE.

To assess the performance of the DDPM and VAE model more closely, this study employed the VAE as a reference approach, which integrates autoencoder with variational inference methods, enabling it to learn latent representations of data and generate new samples. The results are shown in Fig. 8. Sequence logos indicate that promoters synthesized by the VAE mimic natural promoters in the −10 and −35 regions, although significant noise is evident elsewhere. From the position distribution of 6-mer frequency, promoters generated by VAE display consistency with natural promoters in the “TATAAT” and “TAAAAT.” However, an unexpected peak for “ATTATT” is notable in the −30 region of synthetic promoters. Other 6-mers also show some noise in their distribution. This noise may arise due to the VAE model overfitting to certain patterns during training, resulting in the generation of these motifs in biologically non-relevant locations. However, the promoters synthesized by DDPM have a structure and distribution more similar to those of natural promoters, particularly in the key −35 and −10 regions, as shown in Fig. 8. In Fig. 8A, we observe that the DDPM-generated promoters exhibit a clearer alignment with the natural sequence logos, capturing the distinct motifs in these regulatory regions with lower noise levels. Furthermore, as shown in the 6-mer frequency plots in Fig. 8B, the DDPM model produces distributions that closely follow those of natural promoters, while the VAE often generates additional and spurious peaks. This difference suggests that the DDPM model has a better capacity for capturing the positional specificity of promoter motifs, thus leading to more biologically accurate promoter sequences. Indeed, the DDPM outperforms the VAE in promoter design, producing synthetic promoters that more closely mirror the biological characteristics of natural promoters.

### *E. coli* promoter strength prediction based on the transformer

**Fig 9:**
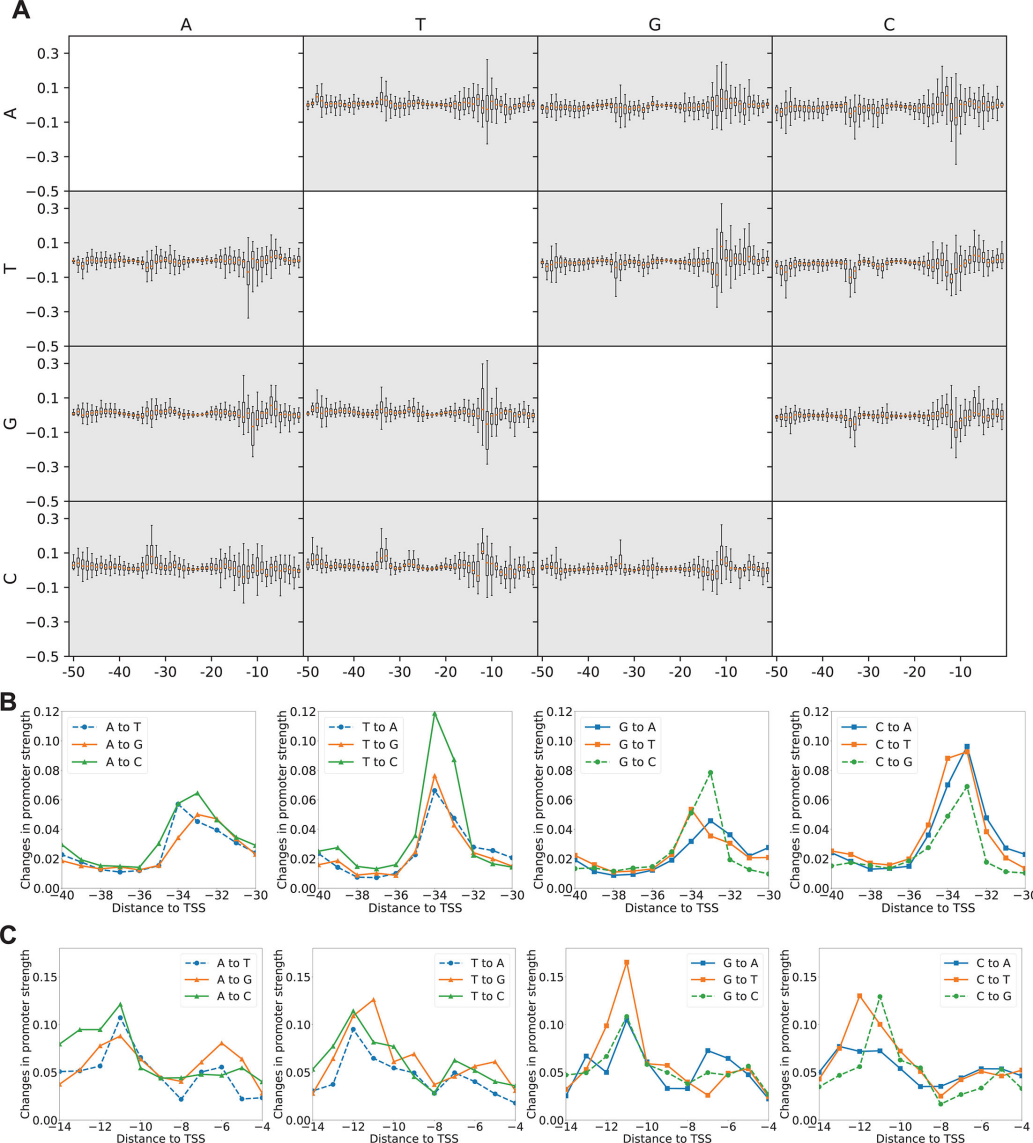
Changes in promoter strength before and after mutation. (**A**) The effect of base mutation on promoter strength. The *x*-axis indicates the location of the mutation, and the *y*-axis indicates the changes in promoter strength before and after the mutation. The left side “A, T, C, G” represents the type of nucleotide before mutation, and the top “A, T, C, G” indicates the type of nucleotide after mutation. Box plots are used to statistically represent the impact of mutations on promoter strength since multiple promoters may undergo the same mutation. (**B**) The changes in promoter strength when the two types of mutations occur in the −35 region. The *x*-axis represents the position of the mutation. The *y*-axis value is obtained by taking the absolute value of the box plot data at the corresponding position in panel **A** and then averaging it. The first type of mutation is represented by the dashed line, and the second type is represented by the solid line. (**C**) The changes in promoter strength when the two types of mutations occur in the −10 region.

After generating synthetic promoters, we further trained a transformer-based predictive model to accurately predict promoter strength. The data set, which consists of *E. coli* natural promoters and their corresponding strength information, was processed using one-hot encoding and then divided into a training set and a testing set with a ratio of 4:1. Given the broad spectrum of promoter strengths, direct normalization could mask the characteristics of weaker promoters. Consequently, we first transformed the promoter strengths using a logarithmic function before applying min-max normalization (see “Data processing”). This approach ensured that the features of all promoter sequences were accurately represented. In this study, a CNN, a type of deep neural network commonly used in image processing and pattern recognition, was applied for the first time to predict promoter strength, achieving a PCC of 0.25 between predicted and actual values. PCC of 0.25 indicates a moderate correlation between the predicted promoter activity and the dRNA-seq levels in the testing set (20). This result is consistent with previous reports for promoter identification and strength prediction (42–44). For the promoter identification task, Shujaat et al. (45) employed CNN to differentiate between promoters and non-promoters. Furthermore, Coppens et al. (46) classified promoters by strength and used CNN to predict promoter strength level. To further improve the performance of the predictive model, we optimized both the encoding method and the prediction model. One-hot encoding fails to capture the correlations between different nucleotides, and it also inadequately addresses position information. Therefore, for enriching the input features of the predictive model, the encoding method illustrated in Fig. 1 was used to extract sequence information, dinucleotide information, and position information from the promoters. Given the limited complexity of CNN, we constructed a transformer-based prediction model in this study. *In silico* experiments demonstrate improved PCC between the predicted and actual values of 0.295. The improvement in the PCC indicates that the strength of synthetic promoters can be predicted more accurately, which allows experimental biologists to utilize more precise promoter information, facilitating a more efficient search for high-performance promoters. To further assess the effectiveness of this predictive model, we investigated the regions sensitive to affecting promoter strength. We randomly selected 200 promoter sequences from the test set and introduced three types of random mutations (for example, if the nucleotide at the current position was A, it was mutated to T, C, or G). Subsequently, we calculated the change in promoter strength before and after the mutation. The results are presented in Fig. 9. Overall, mutations at any position can influence promoter strength, suggesting that nucleotides in any region may contribute to promoter strength. Interestingly, there is a more apparent change in promoter strength when mutations occur in the −10 and −35 regions, corroborating the sequence features of natural promoters (Fig. 6B). Given that the frequencies of A and T are significantly higher than those of G and C in the −10 and −35 regions, we categorized the mutations into two types. The first type encompasses mutual mutations between A and T, as well as between G and C. The second type consists of mutations between the A/T and G/C groups. This section details the changes in promoter strength before and after these two types of mutations, as illustrated in Fig. 9B and C. The effects of these mutations on strength are evident in the regions from −10 to −12 and from −32 to −36, specifically aligning with key feature areas on the promoters. Regarding mutation types, the first type (represented by a dashed line) has a relatively smaller impact on promoter strength compared to the second type (represented by a solid line). These results indicate that our transformer-based model effectively predicts promoter strengths, largely due to its capability to capture essential sequence features, such as those in the −10 and −35 regions.

## DISCUSSION

To summarize, this study leverages advancements in DDPM to synthesize promoters and employs a transformer model to identify high-performance candidates. The analysis shows that the synthetic promoters exhibit features similar to natural promoters in terms of sequence logos, k-mer frequency correlation, and position distribution of the 6-mer frequency. Visualization results of the DDPM training process reveal that the model initially learns features in the −10 region before those in the −35 region. In the promoter strength prediction task, we validated the transformer model performance. We found that mutations in the −10 and −35 regions had a more significant impact on promoter strength than mutations in other regions. Additionally, mutations between A/T and C/G have a significant impact on promoter strength in line with the predictive performance. Overall, this study addresses the challenge of the low proportion of biologically active promoter sequences in the sequence space and the difficulty of searching for them. By utilizing deep learning to extract latent features of promoter sequences, we have successfully synthesized and selected promoters, thus providing a scalable approach to enrich the high-performance promoter library available for experimental validation. Recently, diffusion models have shown superior performance in various fields. Researchers have applied them to interpret medical imaging (47, 48) and design proteins (49). Based on our exploration, DDPM also demonstrated better performance in generating synthetic promoters than VAE. DDPM restores random noise to promoter data through a series of denoising operations. This process is analogous to evolution, where organisms adapt to survive in changing environments. Similarly, the DDPM continuously performs denoising operations with the goal of generating promoters. The VAE is a deep generative model that leverages an encoder-decoder architecture to learn latent data representations. The encoder projects input data into a latent space, where each point signifies a potential compressed form of the input. In this latent space, the VAE captures a distribution over the data rather than a single representation, enabling data augmentation through sampling. The decoder then reconstructs data from these latent representations. By utilizing a “wide-narrow-wide” architecture, the VAE restricts the learned representations to a lower-dimensional space, enhancing data generation and augmentation via variational inference. Although VAE is underpinned by a robust mathematical framework and is prevalent in synthetic biology, it requires a complex encoder network for learning data representations. DDPM, devoid of this requirement, streamlines model architecture and reduces training complexity, thereby enhancing efficiency in processing promoter sequences. Through its diffusion-based sampling, DDPM produces high-quality samples, circumventing the common issues of blurred outputs or mode collapse associated with VAE. This study presents several areas for improvement. Predicting promoter strength is a supervised regression task in which promoter sequences and other relevant features are inputs, and promoter strength is the output. Supervised learning requires a labeled data set, specifically one with known promoter strength values. In general, a larger and higher quality data set improves the predictive model’s performance. However, measuring promoter strength remains a complex and costly experimental task. Therefore, in the future, high-throughput experiments could be combined with deep generative models to address this challenge. Additionally, since the input features of the models may not capture all biological characteristics of promoter sequences, employing natural language processing techniques to develop word vectors from the promoter library could enhance the input features of generative models, thereby improving promoter synthesis technology (50).

Overall, this study utilized DDPM to accomplish the task of synthesizing *E. coli* promoters and employed a transformer-based model for screening high-performance promoters. Our findings present a new case study where deep learning holds significant potential for applications in synthetic biology.

## Data Availability

The implementation of this study is accessible through the GitHub repository at https://github.com/LX2004/promoter. To support further research building on this study’s findings, we have developed an interactive platform that integrates functionalities for promoter generation and strength prediction. This platform allows users to efficiently generate promoters and predict their strengths for multiple species using straightforward operations. The platform can be accessed at https://bioinformatics-syn.org/. The supplemental data of this study are accessible through the GitHub repository at https://github.com/LX2004/promoter.

## SUPPLEMENTAL MATERIAL

File S1 msystems.00183-25-s0001.xlsx The cyanobacteria promoter data set.

File S2 msystems.00183-25-s0002.txt The *Escherichia coli* promoter data set.

File S3 msystems.00183-25-s0003.pdf k-mer correlation coefficients.

Legends msystems.00183-25-s0004.pdf Legends for Files S1 to S3.

ASM does not own the copyrights to Supplemental Material that may be linked to, or accessed through, an article. The authors have granted ASM a non-exclusive, world-wide license to publish the Supplemental Material files. Please contact the corresponding author directly for reuse.
