# Supplementary figures and images for: Combining diffusion and transformer models for enhanced promoter synthesis and strength prediction in deep learning
Source: mSystems. 2025 Mar 19;10(4):e00183-25. doi: 10.1128/msystems.00183-25 (PMC12013266; doi:10.1128/msystems.00183-25)

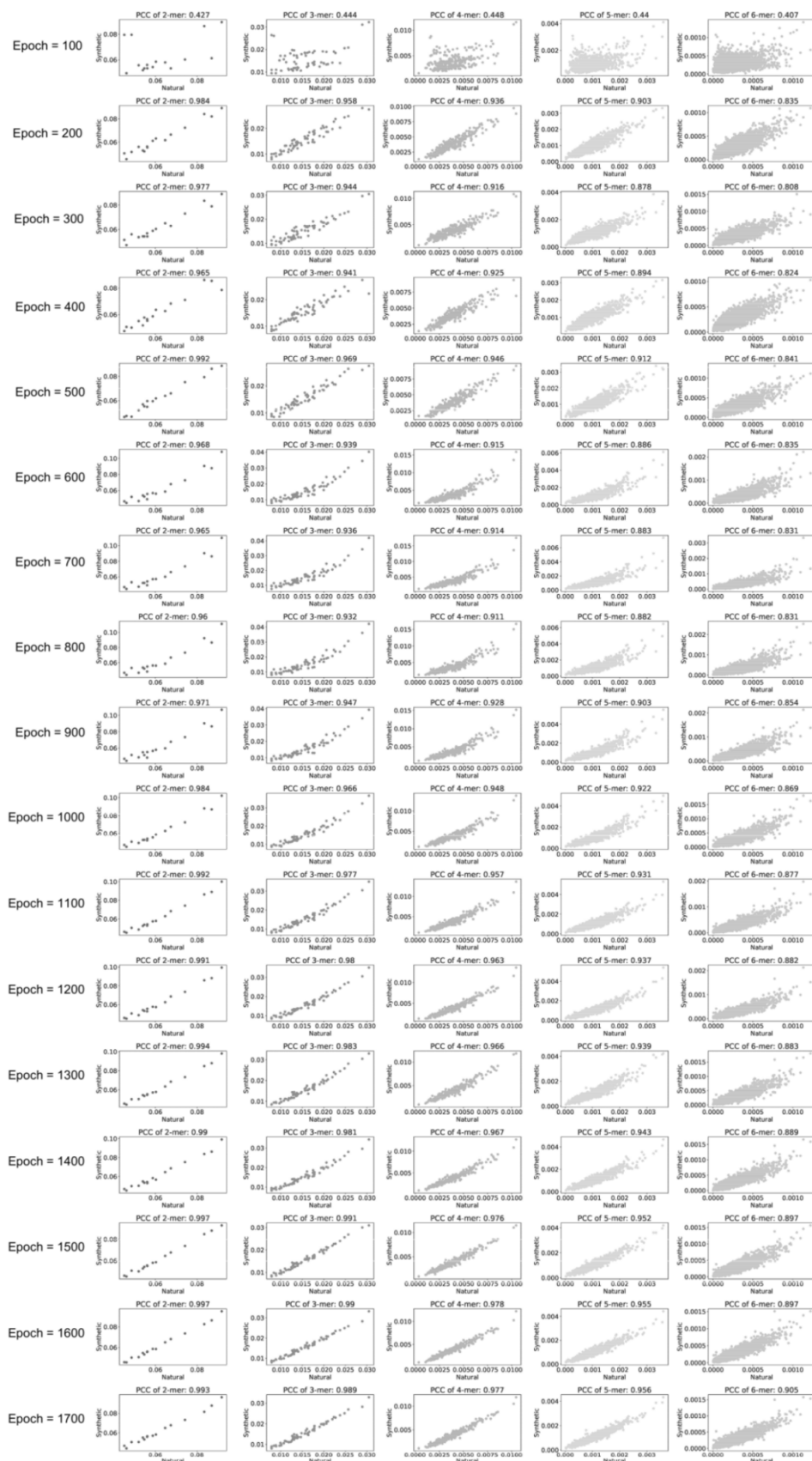

Figure s1. k-mer correlation coefficients at different training periods (k=2~6).

Supplement: File S3 — k-mer correlation coefficients. [file msystems.00183-25-s0003.pdf]
