# Supplementary material for: Combining diffusion and transformer models for enhanced promoter synthesis and strength prediction in deep learning
Source: mSystems. 2025 Mar 19;10(4):e00183-25. doi: 10.1128/msystems.00183-25 (PMC12013266; doi:10.1128/msystems.00183-25)
Supplement: Legends — for Files S1 to S3. [file msystems.00183-25-s0004.pdf]

Combining Diffusion and Transformer Models for Enhanced Promoter Synthesis and Strength Prediction in Deep Learning

SUPPLEMENTARY MATERIAL LEGENDS

'File S1'. The *cyanobacteria* promoter dataset used in this study contains 3,713 samples. Each sample includes a 100-nt long promoter sequence and the strength information of the promoter sequence. The promoter sequences and intensity information are stored in the 'Promoter' and 'Reads' columns, respectively.

'File S2' The *Escherichia coli* promoter dataset used in this study contains 11,884 samples. "Promoter": represents promoter sequences with a length of 50-nt. "Strength": represents the strength of the promoter sequences.

'File S3' A figure about the k-mer correlation coefficients at different training periods (k=2~6).
